# Are aged pTreg cells “the more the better”?

**DOI:** 10.18632/aging.204454

**Published:** 2022-12-22

**Authors:** Weikan Wang, Rachel Thomas, Dong-Ming Su

**Affiliations:** 1 Current affiliation: Department of Neurology, Shanghai Ninth People's Hospital, Shanghai Jiao Tong University School of Medicine, China; 2 Current affiliation: Alcon Research, LLC, Fort Worth, TX 76134, USA; 3 Department of Microbiology, Immunology, and Genetics, University of North Texas Health Science Center, Fort Worth, TX 76107, USA

**Keywords:** neuroinflammation, aged pTreg accumulation, infiltration obstruction, transient pTreg inhibition, amelioration

The driving force behind age-related neuroinflammatory disorders is primarily associated with the aberrant aged immune system, particularly T cell-associated cellular immunity. The T cells participating in such disorders include pathogenic T effector (Teff) and protective regulatory T (Treg) cells. Generally, FoxP3^+^CD4^+^ Treg cells play an ameliorative role in age-related neuroinflammatory disorders’ onset and severity [1]. However, there are distinct functions (suppressing inflammation or inhibiting immunity) performed by these Treg cells, depending on their locality, such as residing in peripheral lymphoid organs or in certain tissues (termed tissue-resident Treg) [2].

The CNS is considered immune privileged, and generally it is isolated from the lymphatic system by specialized membranes, including the blood-brain barrier (BBB) and choroid plexus (CP). This may result in different proportions of T cell distribution inside and outside of the CNS. Recently work found that distributions of young and aged polyclonal/pan- Treg cells in the peripheral lymphoid organs, termed pTreg, and the central nervous system (CNS), termed CNS-Treg, during the autoimmune neuroinflammation were different. The percentages of pTreg cells were lower in the inflamed aged CNS, but higher in the aged peripheral lymphoid organs, compared to young conterparts [3].

Age-related neuroinflammatory disorders, such as Alzheimer’s disease (AD), are commonly seen in the elderly. Although T cell-mediated autoimmune multiple sclerosis (MS) disease typically has a young adults (20~40 years old) onset, late-onset MS in the elderly (diagnosis at the age of 60 or older) has been increasedly reported, and the aged MS patients have a more severe progressive course, for example 29% with primary progressive (without remission) and 26% with second progressive (with irreversible damage and disability) MS. Of the milder relapsing-remitting MS progression, there are far fewer late-onset MS patients (<40%, compared to their younger counterparts, of whom account for >80% of the milder progression) [4].

Ample evidence indicates that aging results in relatively enhanced generation of thymic Treg (tTreg) [5] and greatly accumulated pTreg in the peripheral lymphoid organs [6]. Intriguingly, the accumulated and increased pTreg cells in the aged immune system fail to ameliorate severe MS disease in the elderly. To uncover why, recently, Wang et al. [3] used a mouse model of MS, experimental autoimmune encephalomyelitis (EAE), to transiently (Treg recovery occurs 2 weeks after the inhibition) suppress FoxP3 expression in accumulated pan-pTreg cells in the aged mice and this inhibition results in a modest improvement in the EAE illness in the aged animals, and increased proportion of CNS-Treg cells in the inflamed aged CNS. Inhibition of these pTreg cells was achieved by two methods, using either pharmacological P300i on wild-type aged mice or diphtheria toxin (DT) on aged FoxP3-DTR (diphtheria toxin receptor) transgenic mice. These two methods can impair Treg suppressive activities by inhibiting FoxP3 expression or abolishing pTreg cell responses without affecting Teff cell responses. A similar effect was also observed by depletion of pTreg cells in AD disease, resulting in mitigation of the neuroinflammatory response, reversal of cognitive decline, and reduction of amyloid-beta plaques [7].

**Figure 1:**
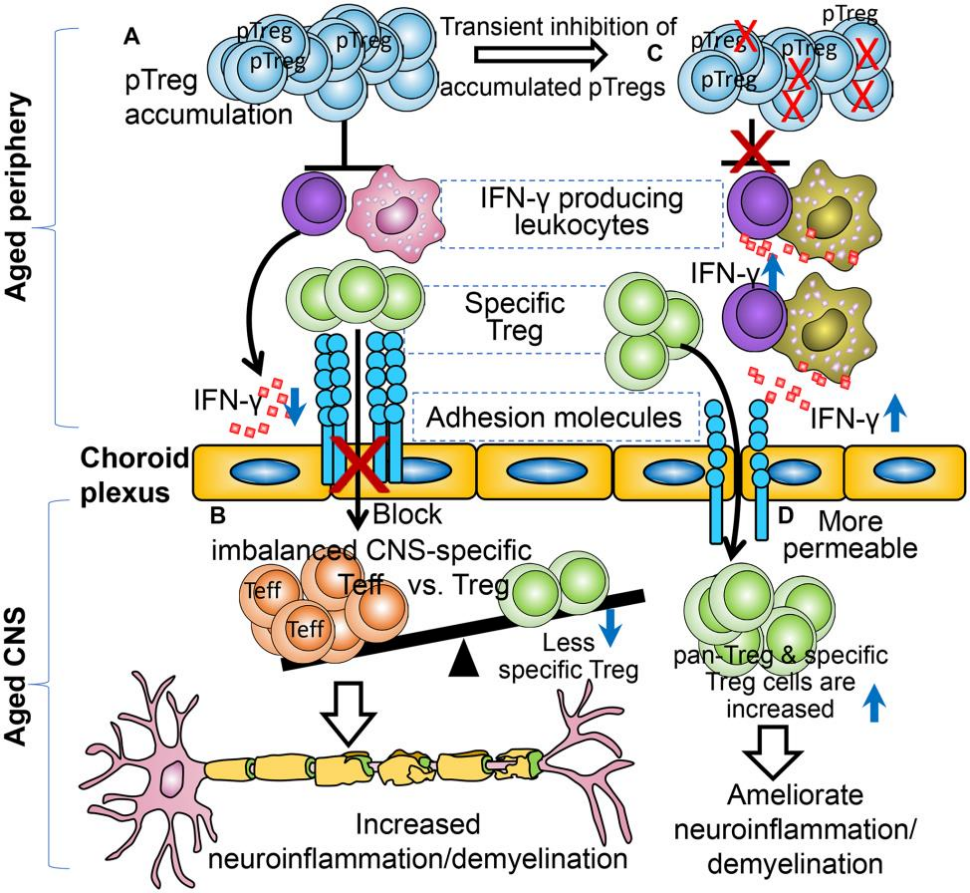
**Illustration of the participation of aged excessive pTreg cells in an age-related neuroinflammatory disorder.** (**A**) Aged mice have accumulated peripheral Treg cells but display more severe EAE symptoms, due to over-suppression of IFNγ producing leukocytes. (**B**) Accumulated peripheral Tregs impair immune cells, including specific Treg cells, the infiltration into the CNS (red “X”), resulting in imbalanced CNS Teff versus Treg cells. (**C**) Transient inhibition of accumulated and excessive peripheral Tregs (right top red “Xs”). (**D**) The CNS-infiltration pathway is more permeable when the peripheral Treg cells are transiently inhibited, and more CNS-Treg cells are detected and the EAE disease is ameliorated in the aged inflamed CNS.

The underlying mechanism is hypothesized to be that in the elderly, accumulated and excessive pTreg cells residing in the BBB and brain CP (Figure 1A) potentially hamper trafficking of monocytes and antigen-specific Treg cells into the inflamed CNS (Figure 1B). This blockade is due to an excessive suppression of IFNγ-secreting leukocytes, as we know that IFNγ promotes CNS infiltration of immune cells [8]. Once the excessive pTreg cells are transiently inhibited (Figure 1C), INFγ-expression is increased in the CP-adhering CD45^+^ (hematopoietic lineage) cells and the BBB and CP are become more permeable for immune cell infiltration (Figure 1D) [3].

The studies from these two groups in EAE [3] and AD [7] models offer strong evidence that accumulated and functionally compromised aged pTreg cells may not be beneficial, but rather may play a detrimental role in the course and severity of age-related neuroinflammatory disorders. In conclusion, lack of Treg cells is detrimental, however, during age-related neuroinflammatory disorders, CNS-resident Treg cells are likely considered “the more the better”, but peripheral Treg cells are likely not.
